# Prophylactic Antibiotics for Endoscopy-Associated Peritonitis in Peritoneal Dialysis Patients

**DOI:** 10.1371/journal.pone.0071532

**Published:** 2013-08-01

**Authors:** Hsin-Hsu Wu, I-Jung Li, Cheng-Hao Weng, Cheng-Chia Lee, Yung-Chang Chen, Ming-Yang Chang, Ji-Tseng Fang, Cheng-Chieh Hung, Chih-Wei Yang, Ya-Chung Tian

**Affiliations:** 1 Kidney Research Center, Department of Nephrology, Lin-Kou Chang Gung Memorial Hospital, Taiwan and Chang Gung University, Tao Yuan, Taiwan; 2 Graduate Institute of Clinical Medical Sciences, Chang Gung University, Tao Yuan, Taiwan; University of Sao Paulo Medical School, Brazil

## Abstract

**Introduction:**

Continuous ambulatory peritoneal dialysis (CAPD) peritonitis may develop after endoscopic procedures, and the benefit of prophylactic antibiotics is unclear. In the present study, we investigated whether prophylactic antibiotics reduce the incidence of peritonitis in these patients.

**Patients and methods:**

We retrospectively reviewed all endoscopic procedures, including esophagogastroduodenoscopy (EGD), colonoscopy, sigmoidoscopy, cystoscopy, hysteroscopy, and hysteroscopy-assisted intrauterine device (IUD) implantation/removal, performed in CAPD patients at Chang Gung Memorial Hospital, Taiwan, between February 2001 and February 2012.

**Results:**

Four hundred and thirty-three patients were enrolled, and 125 endoscopies were performed in 45 patients. Eight (6.4%) peritonitis episodes developed after the examination. Antibiotics were used in 26 procedures, and none of the patients had peritonitis (0% vs. 8.1% without antibiotic use; p = 0.20). The peritonitis rate was significantly higher in the non-EGD group than in the EGD group (15.9% [7/44] vs. 1.2% [1/81]; p<0.005). Antibiotic use prior to non-EGD examinations significantly reduced the endoscopy-associated peritonitis rate compared to that without antibiotic use (0% [0/16] vs. 25% [7/28]; p<0.05). Peritonitis only occurred if invasive procedures were performed, such as biopsy, polypectomy, or IUD implantation, (noninvasive procedures, 0% [0/20] vs. invasive procedures, 30.4% [7/23]; p<0.05). No peritonitis was noted if antibiotics were used prior to examination with invasive procedures (0% [0/10] vs. 53.8% [7/13] without antibiotic use; p<0.05). Although not statistically significant, antibiotics may play a role in preventing gynecologic procedure-related peritonitis (antibiotics, 0% [0/4] vs. no antibiotics, 55.6% [5/9]; p = 0.10).

**Conclusion:**

Antibiotic prophylaxis significantly reduced endoscopy-associated PD peritonitis in the non-EGD group. Endoscopically assisted invasive procedures, such as biopsy, polypectomy, IUD implantation/removal, and dilatation and curettage (D&C), pose a high risk for peritonitis. Prophylactic antibiotics for peritonitis prevention may be required in colonoscopic procedures and gynecologic procedures.

## Introduction

Peritonitis is the most common cause of technique failure and dropout from continuous ambulatory peritoneal dialysis (CAPD) therapy. It can lead to serious complications, even death. The outcomes of peritonitis caused by different microorganisms are diverse. Patients with peritonitis caused by gram-negative microorganisms have worse outcomes, including recurrent peritonitis and technique failure, compared to that in patients with peritonitis caused by coagulase-negative Staphylococcal species or *Staphylococcus aureu* [1], [2].

Several paths leading to peritonitis in peritoneal dialysis (PD) patients have been suggested, including intraluminal, periluminal, hematogenous, transmural, and ascending routes [3]. The transmural migration of bacteria from the bowel lumen into the peritoneal cavity, which is an important source of peritonitis, has been verified in animals [4], [5]. These studies recommend consideration of the occurrence of peritonitis during any manipulation of the gastrointestinal tract. Several reports have demonstrated a link between colonoscopy and the occurrence of PD peritonitis [6], [7], [8], [9], [10], [11]. Both diagnostic and therapeutic endoscopic procedures precipitate PD peritonitis. Perioperative antibiotics were shown to be effective for preventing surgery-associated peritonitis [12], [13]. However, the benefit of prophylactic antimicrobial agents in preventing PD peritonitis in patients receiving gastrointestinal examinations is not well-documented. Yip et al. showed that the risk of peritonitis reduced after colonoscopy if prophylactic antibiotics were used despite no statistical significance [7].

In addition to the transmural route, the ascending route by which microorganisms migrate from the female reproductive tract into the peritoneal cavity to cause peritonitis has been demonstrated in PD patients receiving hysteroscopy [14], [15], [16]. Despite acknowledgement of the importance of hysteroscopy-associated PD peritonitis, it is unknown whether prophylactic antibiotics reduce the occurrence of posthysteroscopy peritonitis.

In the present study, we investigated the risk of PD peritonitis after endoscopic procedures, including esophagogastroduodenoscopy (EGD), colonoscopy, sigmoidoscopy, cystoscopy, and hysteroscopy. Furthermore, we attempted to determine whether prophylactic antibiotics prevented the occurrence of endoscopy-associated peritonitis in PD patients.

## Patient and Methods

This study enrolled 433 patients who were receiving PD dialysis at Linkou Chang Gung Memorial Hospital, a 3000-bed tertiary medical center in northern Taiwan, in February 2012. We retrospectively reviewed all endoscopic procedures, including EGD, small bowel enteroscopy, colonoscopy, sigmoidoscopy, hysteroscopy, hysteroscopy-assisted intrauterine device (IUD) implantation/removal, and dilatation and curettage (D&C), performed in the PD patients between February 2001 and February 2012. Demographic data, including age, gender, serum albumin, creatinine, phosphate, uric acid levels, the duration of PD dialysis, and underlying comorbidities, including diabetes mellitus, liver cirrhosis, and chronic viral hepatitis, were recorded. Endoscopy-associated peritonitis was defined as the occurrence of peritonitis within 24 h after examination. The diagnosis of PD peritonitis was made by the presence of cloudy effluent dialysate, abdominal pain with or without fever, and more than 100/mm^3^ of white blood cells with over 50% polymorphonucleocytes in peritoneal effluent over 2 h. The use of oral or intravenous antibiotics prior to the procedures was also recorded. These included prophylactic antibiotics intended to prevent endoscopy-associated PD peritonitis and antibiotics used for the treatment of infections such as pneumonia and urinary tract infection (UTI).

Statistical analysis was performed using IBM SPSS Statistics version 20 (IBM USA) for Windows. Data were expressed as mean ± standard deviation. A p-value of <0.05 was considered statistically significant. All probabilities were 2-tailed. The chi-square test, Fisher’s exact test, Student’s *t*-test, and multiple logistic regression analysis were used as appropriate.

Written informed consents were obtained from all patients and this study was approved by the institutional review board of Chang Gung Memorial Hospital, Taiwan.

## Results

### Potential Advantage of Prophylactic Antibiotics in Reducing the Incidence of Endoscopy-associated PD Peritonitis

A total of 433 patients who were receiving PD dialysis in February 2012 were enrolled in this study. Among these patients, 45 patients underwent 125 endoscopic procedures between February 2001 and February 2012, including EGDs, small bowel enteroscopies, colonoscopies, sigmoidoscopies, cystoscopies, and hysteroscopies. The mean age was 47±12 years (range, 24–86 years), and the mean dialysis duration was 5.2±3.4 years (range, 0.9–15 years). The incidence of endoscopy-associated PD peritonitis (defined as the occurrence of peritonitis within 24 h after examination) was 6.4% (n = 8). Patients with endoscopy-associated peritonitis had higher white blood cell counts, higher serum C-reactive protein levels, and lower serum calcium level than patients without peritonitis (Table 1). Different microorganisms were isolated from the effluent dialysates of the PD peritonitis patients (Table 2). To assess whether antibiotic prophylaxis prior to endoscopic examination was able to reduce endoscopy-associated PD peritonitis, the use of antibiotics in 125 endoscopic procedures was analyzed. The data demonstrated that antibiotics were administered prior to the endoscopic procedures in only 26 procedures (20.8%), including prophylactic antibiotics intended to prevent peritonitis or endocarditis (n = 21, 80.8%) and antibiotics used for the treatment of pneumonia, UTI, or biliary tract infection (n = 5, 19.2%) (Table 3). No patients who took antibiotics prior to endoscopic procedures developed PD peritonitis. In contrast, without prophylactic antibiotics, 8 episodes of endoscopy-associated PD peritonitis occurred in 99 endoscopic procedures (8.1%). Despite no statistically significant difference in the prevention of endoscopy-associated PD peritonitis using prophylactic antibiotics (antibiotic use, 0% vs. no antibiotic use, 8.1%, p = 0.20), antibiotic use prior to endoscopic procedures had a tendency to reduce the incidence of postendoscopic PD peritonitis.

**Table 1:** Demographic Data of PD Patients who Underwent Endoscopic Procedures.

	No peritonitis (n = 117)	Peritonitis (n = 8)	p-value
Age	47±12	47±9	NS
Dialysis duration (years)	5.26±3.40	4.07±3.45	NS
Hemoglobin (g/dL)	10.04±1.62	9.10±1.92	NS
White blood cell count (1000/µL)	7.76±2.88	11.66±4.64	<0.05
Albumin (g/dL)	3.65±0.63	3.64±0.68	NS
C-reactive protein (mg/L)	14.96±15.70	45.49±65.47	<0.001
Creatinine (mg/dL)	12.75±3.00	12.35±2.30	NS
Calcium (mg/dL)	9.97±0.99	9.13±0.46	<0.05
Phosphorus (mg/dL)	5.55±1.63	6.25±1.78	NS
Uric acid (mg/dL)	7.20±0.98	7.77±1.03	NS
Total cholesterol (mg/dL)	195.83±35.86	218.71±26.87	NS
Diabetes mellitus	5 (4.3%)	0 (0%)	NS
HBV infection	35 (29.9%)	3 (37.5%)	NS
HCV infection	9 (7.7%)	0 (0%)	NS
Antibiotic use	26 (22.2%)	0 (0%)	NS

HBV = Hepatitis B virus, HCV = Hepatitis C virus.

**Table 2:** Bacterial Species that Caused Endoscopy-associated Peritonitis.

	Procedures	Dialysate culture
1	EGD+local hemostasis	*E*. *coli*
2	Colonoscopy+polypectomy	*E*. *coli*+*Klebsiella pneumoniae*+*Enterococcus faecalis*
3	Colonoscopy+polypectomy	Culture negative
4	Hysteroscopy+currettage	Culture negative
5	Hysteroscopy+polypectomy	Culture negative
6	Hysteroscopy+IUD implantation	*Enterococcus faecalis*
7	Hysteroscopy+IUD implantation	*Streptococcus* Gr. B
8	Hysteroscopy+IUD implantation	*Streptococcus* Gr. D+*E*. *coli*

EGD = Esophagogastroduodenoscopy, IUD = Intrauterine device.

**Table 3:** Antibiotics Used Prior to Endoscopic Procedures.

Case	Procedures	Antibiotics	Indication	
1	Colonoscopy+polypectomy	Ampicillin+gentamicin+metronidazole		Treatment of biliary tract infection
2	Colonoscopy+polypectomy	Vancomycin+ceftazidime		Treatment of pneumonia
3	Colonoscopy	ceftriaxone		Prophylaxis
4	Colonoscopy	Ceftriaxone		Prophylaxis
5	Colonoscopy	Ceftriaxone		Prophylaxis
6	Colonoscopy+local hemostasis	Ceftriaxone		Prophylaxis
7	Cystoscopy	Oral cefadroxil		Prophylaxis
8	Cystoscopy	Ampicillin		Prophylaxis
9	Cystoscopy+biopsy	Cefazolin		Prophylaxis
10	Cystoscopy+retrograde pyelogram	Oral cefadroxil		Prophylaxis
11	Cystoscopy+retrograde pyelogram	Oral sulfamethoxazole+trimethoprim		Prophylaxis
12	Dilatation and curettage	Oral clindamycin		Prophylaxis
13	Dilatation and curettage	Oral cefadroxil		Prophylaxis
14	Dilatation and curettage	Oral clindamycin		Prophylaxis
15	Hysteroscopy	Oral clindamycin		Prophylaxis
16	Transrectal prostate biopsy	Oral levofloxacin		Prophylaxis
17	EGD	Cefazolin		Treatment of urinary tract infection
18	EGD	Ceftriaxone+vancomycin		Treatment of pneumonia
19	EGD	Ceftriaxone		Treatment of pneumonia
20	EGD	Oral ceftibuten		Prophylaxis
21	EGD+biopsy	Augmentin		Prophylaxis
22	EGD+hemostasis	Oral cefadroxil		Prophylaxis
23	EGD+local hemostasis	Oral ceftibuten		Prophylaxis
24	EGD+local hemostasis	Oral ceftibuten		Prophylaxis
25	EGD+local hemostasis	Oral ceftibuten		Prophylaxis
26	EGD+polypectomy	Oral ceftibuten		Prophylaxis

EGD = Esophagogastroduodenoscopy.

### Reduction of Non-EGD Endoscopy-associated Peritonitis with Prophylactic Antibiotic Use

Compared with colonoscopy and endoscopy of the small intestine that have been shown to cause PD peritonitis, EGD has not been reported to increase the risk of transmural peritonitis in PD patients. We therefore subclassified the endoscopic procedures into the EGD group and the non-EGD group. The non-EGD group included colonoscopy, sigmoidoscopy, small intestine enteroscopy, cystoscopy, hysteroscopy, and hysteroscopy-assisted implantation or removal of IUDs. In the EGD group, a total of 81 EGD procedures were performed, including 46 procedures of EGD alone and 35 procedures of EGD with other endoscopic invasive procedures, including endoscopic biopsy, esophageal variceal ligation, and endoscopy-assisted local hemostasis. Although no prophylactic antibiotics were used in these 81 procedures, only 1 patient (1.2%) developed PD peritonitis after EGD with endoscopic local hemostasis for duodenal ulcer bleeding. The result suggested a low incidence of PD peritonitis after EGD.

In the non-EGD group, a total of 43 procedures were analyzed. The incidence of endoscopy-associated PD peritonitis in the non-EGD group was significantly higher than in the EGD group (15.9% [7/44] vs. 1.2% [1/81]; p<0.05) (Table 4). Interestingly, antibiotics were used prior to 16 non-EGD procedures, and no PD peritonitis occurred after these procedures. In contrast, among 28 non-EGD procedures without prophylactic antibiotics, 7 episodes of endoscopy-associated PD peritonitis (25%) developed (Table 4). Antibiotic use prior to non-EGD procedures significantly reduced the endoscopy-associated PD peritonitis rate (0% vs. 25% without antibiotic use; p<0.05).

**Table 4:** Effect of Prophylactic Antibiotics on the Incidence of Endoscopy-associated Peritonitis.

Group	Peritonitis (%)
EGD (n = 81)	1 (1.2%)
Prophylactic antibiotics (n = 10)	0 (0%)
No prophylactic antibiotics (n = 71)	1 (1.4%)
Non-EGD (n = 44)	7 (15.9%)^a^
Prophylactic antibiotics (n = 15)	0 (0%)^b^
No prophylactic antibiotics (n = 28)	7 (25%)

EGD = Esophagogastroduodenoscopy.

a Peritonitis rate was significantly higher in the non-EGD group compared with the EGD group, p<0.05.

b Antibiotic use prior to non-EGD procedures significantly reduced the endoscopy-associated PD peritonitis rate, p<0.05.

Because endoscopy with invasive therapies is more likely to result in bowel perforation and transmural infection compared with endoscopy without invasive therapies, we further analyzed the effect of invasive procedures on the occurrence of PD peritonitis in the non-EGD group. The data showed that PD peritonitis did not occur after any of the procedures (colonocopy, sigmoidoscopy or hysteroscopy) without invasive therapies, whereas 7 episodes of PD peritonitis developed after these endoscopic procedures in combination with endoscopic colon biopsies, colonic polypectomy, or IUD implantation (no invasive therapy, 0% [0/20] vs. invasive therapy, 30.4% [7/23]; p<0.05). Whether prophylactic antibiotics reduced the incidence of PD peritonitis after these invasive therapies was further investigated. The results demonstrated that the rate of PD peritonitis after these invasive therapies without prophylactic antibiotics was significantly higher than in cases with antibiotic use prior to the therapies (antibiotics, 0% [0/10] vs. no antibiotics: 53.4% [7/13]; p<0.005) (Table 5).

**Table 5:** Effect of Prophylactic Antibiotics on the Incidence of Endoscopy-associated Peritonitis in Patients Undergoing Non-EGD Procedures with Invasive Therapies.

Antibiotic use	Peritonitis
Invasive therapies (n = 23)	7 (30.4%)
No prophylactic antibiotics (n = 13)	7 (53.8%)
Prophylactic antibiotics (n = 10)	0 (0%)^a^

a Antibiotic use prior to the non-EGD procedures with invasive therapies significantly reduced the endoscopy-associated PD peritonitis rate, p<0.05.

We also analyzed gynecologic procedures and their relationship to peritonitis in CAPD patients. Thirteen gynecologic procedures were performed in our cohort, including hysteroscopy, IUD implantation or removal, and D&C. Five peritonitis episodes were noted after gynecologic procedures, and the peritonitis rate was significantly higher than other groups (gynecologic procedures, 38.5% [5/13] vs. colonoscopy and small bowel enteroscopy, 7.7% [2/26]; p<0.05). Four patients received antibiotics before these procedures and none of those 4 patients (0%) developed PD peritonitis, whereas 5 of 9 patients (55.6%) without prophylactic antibiotic use developed PD peritonitis. Although the results did not reach statistical significance, no peritonitis occurred if antibiotics were used prior to these gynecologic exams (0% [0/4] vs. 55.6% [5/9] without antibiotic use; p = 0.10) (Table 6).

**Table 6:** Effect of Prophylactic Antibiotics on the Incidence of Endoscopy-associated Peritonitis in Patients Undergoing Gynecologic Procedures.

Antibiotic use	Peritonitis
Gynecologic procedures (n = 13)	5 (38.5%)
No prophylactic antibiotics (n = 9)	5 (55.6%)
Prophylactic antibiotics (n = 4)	0 (0%)^a^

a No peritonitis occurred if antibiotics were used prior to these gynecologic exams, p = 0.10.

## Discussion

Transmural migration from the bowel into the peritoneal cavity leading to peritonitis has been demonstrated in animal studies [5]. Any irritation of the bowel that can enhance the transmigration of bacteria across the bowel wall increases the risk of peritonitis. Treatment of constipation using laxatives or enemas may irritate the bowel and facilitate the transmural migration of bacteria, causing peritonitis in PD patients [4]. Endoscopic procedures require inflation of the bowel and can irritate the bowel wall during manipulation, which can enhance the transmural migration of intestinal flora. Colonoscopic procedures have been reported to precipitate transmigration of bacteria across the bowel wall and cause subsequent peritoneal seeding and peritonitis [17]. Although the incidence of endoscopy-associated peritonitis is low, it remains one of the most serious complications of this procedure when it occurs [6].

The risk of endoscopy-associated peritonitis may be even higher in PD patients than in the general population because glucose in the PD dialysate provides a breeding ground for bacterial growth. Additionally, defense mechanisms are jeopardized because of reduced antibacterial opsonization resulting from diluted intraperitoneal cytokines, antibodies, and complement, and dysfunction of the peritoneal mesothelial cells. Our study demonstrated that the incidence of endoscopy-associated peritonitis in PD patients was 6.3%, which was higher than the reported incidence (<0.1%) in non-PD patients in our hospital. This result verifies an increased risk of endoscopy-associated peritonitis in PD patients than in non-PD patients.

Under physiological conditions, swallowed bacteria from the oral cavity or the upper respiratory tract are transiently discovered in the upper gut and remain at low bacterial counts (<10^5^ colony-forming units (CFU)/mL) [18]. In contrast, colonized bacteria counts in the colon commonly exceed 10^9^ CFU/mL. Gastric juice is known to have a bactericidal effect [19]. The low pH in gastric juice, as well as pepsin, underlie this effect. [20] The prevalence of EGD-associated peritonitis is, in theory, lower than colonoscopy-associated peritonitis. Similarly, a large quantity of normal microflora in the vagina (10^5^–10^8^ CFU/mL) [21], [22], which can ascend into the peritoneal cavity through the female reproductive tract during hysteroscopy, increases the risk of the endoscopy-associated peritonitis. Consistent with this theory, our study showed that the incidence of the endoscopy-associated PD peritonitis (1.2%) in the EGD group was significantly lower than that (16.3%) in the non-EGD group. In addition to different colonized bacteria counts in different areas, the difference in the incidence of endoscopy-associated PD peritonitis between the 2 groups may also reflect the possibility of bacterial access into the peritoneal cavity during the endoscopic procedures. Compared with colonoscopy, the transmural migration of bacteria into the peritoneal cavity during EGD is hindered by a greater mural thickness and a shorter bowel segment allowing for transmigration (the lower part of stomach and the upper part of duodenum vs. the entire colon). The ascending route from the female reproductive tract during hysteroscopy may be more accessible for bacteria to enter the peritoneal cavity, as the incidence of hysteroscopy-associated PD peritonitis was significantly higher than that of EGD-associated PD peritonitis.

In the present study, we demonstrated that prophylactic antibiotics reduced the incidence of postendoscopic PD peritonitis. Because the data revealed that EGD seldom caused PD peritonitis, even without prophylactic antibiotic use, we further analyzed the beneficial effect of antibiotic use prior to non-EGD endoscopic procedures in preventing PD peritonitis. Antibiotic use prior to non-EGD procedures significantly reduced endoscopy-associated PD peritonitis (0% vs. 25% without antibiotics). Further analysis revealed that endoscopic procedures with invasive therapies, including endoscopic colon biopsies, colonic polypectomy, or IUD implantation, were a decisive factor for the development of the postendoscopic PD peritonitis. None of the patients receiving colonoscopy or hysteroscopy without invasive therapies developed PD peritonitis, whereas 30.4% of patients receiving these procedures with invasive therapies developed endoscopy-associated PD peritonitis. Prophylactic antibiotics significantly reduced the incidence of PD peritonitis after non-EGD procedures with invasive therapies.

Yip et al. have presented an incidence of 6.3% for postcolonoscopic PD peritonitis and a beneficial effect of prophylactic antibiotics on the prevention of these complications, although the result did not reach statistical significance [7]. The International Society for Peritoneal Dialysis (ISPD) 2010 guidelines for peritonitis had accordingly recommended prophylactic antibiotics in PD patients undergoing colonoscopy [3], [23]. Our study demonstrated an incidence of 6.6% of colonoscopy-associated PD peritonitis (2 episodes of PD peritonitis after 30 colonoscopic procedures). These episodes occurred in patients receiving colonoscopy and polypectomy without prophylactic antibiotics, implying a requirement for antibiotic prophylaxis.

Gynecologic procedures are a rare cause of PD peritonitis, by which vaginal colonized bacteria or fungi may be spread into the peritoneal cavity during the procedure or manipulation [15], [16], [24], [25]. Although prophylactic antibiotics are recommended for the prevention of colonoscopy-associated PD peritonitis in the ISPD 2010 guidelines, the advantage of prophylactic antibiotics in hysteroscopy has not been addressed. Our study showed that 5 patients who received hysteroscopy with prophylactic antibiotics did not develop PD peritonitis, whereas 5 patients among 13 undergoing hysteroscopy without prophylactic antibiotics developed posthysteroscopic PD peritonitis. This result suggested that antibiotic prophylaxis provides a protective effect on the development of PD peritonitis in patients undergoing gynecologic procedures.

The ISPD 2005 peritoneal dialysis-related infection guidelines recommended ampicillin 1 g plus a single dose of an aminoglycoside, with or without metronidazole, given intravenously just prior to patients undergoing colonoscopy with polypectomy to decrease the risk of peritonitis [26]. Various antibiotics were administered prior the endoscopic examinations in this study. One dose of 1g Ceftriaxone administration was used as prophylaxis before colonoscopy and none of the patients had endoscopy-associated PD peritonitis. Considering the normal flora in human gut [27], third generation cephalosporins may be appropriate choices for peritonitis prophylaxis. Gynecologic procedures also carry high risk of endoscopy-associated PD peritonitis. Enterococcal peritonitis and streptococcal peritonitis after hysteroscopy and IUD implantation were noted in our study. It may be appropriate that antibiotic regimen including an agent active against enterococcus and streptococcus. Clindamycin and first generation cephalosporin were used in our series and none of the patients developed peritonitis after these gynecologic examinations. There are several limitations in our study. First, the data were collected retrospectively. Second, this study was conducted in a single tertiary medical center, and endoscopy-associated complications may vary in different hospitals. Third, the study recorded only 128 endoscopic procedures and may underestimate the importance of antibiotic prophylaxis. Therefore, a large randomized trial is required to address the efficacy of antibiotic prophylaxis in the prevention of postendoscopic PD peritonitis. Nevertheless, because endoscopy-associated PD peritonitis only occurred in patients undergoing colonoscopy or hysteroscopy without antibiotic use, and none of the patients receiving prophylactic antibiotics developed postendoscopic PD peritonitis, this study merits the use of antibiotic prophylaxis in patients undergoing colonoscopy and hysteroscopy.

In conclusion, this study demonstrated a low incidence of EGD-associated PD peritonitis. Antibiotic prophylaxis significantly reduced endoscopy-associated PD peritonitis in the non-EGD group. Endoscopically assisted invasive procedures, such as biopsy, polypectomy, IUD implantation/removal, and dilatation and curettage, pose a high risk for peritonitis. Prophylactic antibiotics significantly reduce the rate of PD peritonitis after these invasive therapies.
